# Two-Dimensional Materials: From Synthesis to Applications

**DOI:** 10.3390/molecules30030741

**Published:** 2025-02-06

**Authors:** Sake Wang, Nguyen Tuan Hung, Minglei Sun

**Affiliations:** 1 College of Science, Jinling Institute of Technology, 99 Hongjing Avenue, Nanjing 211169, China; 2 Frontier Research Institute for Interdisciplinary Sciences, Tohoku University, Sendai 980-8578, Japan; nguyen.tuan.hung.e4@tohoku.ac.jp; 3 NANOlab Center of Excellence and Department of Physics, University of Antwerp, Groenenborgerlaan 171, 2020 Antwerp, Belgium; minglei.sun@uantwerpen.be

## 1. Introduction

Two-dimensional materials have become a cornerstone of modern materials science, offering unique structural, electronic, optical, and mechanical properties [1]. This SI of *Molecules* brings together 17 diverse contributions, showcasing the latest advancements in 2D material synthesis, theoretical insights, and applications [2,3]. These papers collectively highlight the transformative potential of 2D materials in energy, environmental sustainability, catalysis, sensors, and more. This editorial provides a detailed analysis and categorization of these works, emphasizing their importance and future implications.

## 2. Synthesis and Characterization: Building the Foundation of 2D Materials

The synthesis of high-quality 2D materials is the first step in realizing their potential. This SI includes three contributions on optimizing synthesis techniques and characterizing the resulting materials to enhance their applicability.

1. Hexagonal boron nitride thin films.
  This study employed high-power impulse reactive magnetron sputtering to deposit hexagonal boron nitride (h-BN) thin films. By tuning deposition parameters such as temperature, nitrogen flow, and time, the team achieved control over crystallinity, surface roughness, and chemical composition. The results provide insights into optimizing h-BN films for electronic and optical devices, underscoring the importance of process parametrization. (Contribution 1).
2. Carbon nitride nanosheets for silica film adhesion.
  A critical challenge in fabricating VMSF is ensuring their stability on carbonaceous electrodes. This study demonstrated that carbon nitride nanosheets act as effective adhesive layers, improving the mechanical stability of VMSF. This enhancement enabled the development of robust immunosensors with broad detection ranges, highlighting the role of hybrid material interfaces. (Contribution 2).
3. GO-based anticorrosion coatings.
  Functionalizing GO with ethylenediamine introduced a sustainable way to enhance waterborne polyurethane coatings’ anticorrosion performance. The low GO content effectively improved the coatings’ barrier properties, offering environmentally friendly solutions for corrosion protection. This study illustrates the potential of GO-based additives for large-scale industrial applications. (Contribution 3).

## 3. Energy Applications: Powering a Sustainable Future

Energy sustainability is one of the most critical challenges of our time. Four contributions in this SI highlight the role of 2D materials in advancing energy conversion and storage technologies.

1. MoS_2_-based broadband solar absorbers.
  A single-layer molybdenum disulfide (MoS_2_) was used to design a broadband solar absorber with exceptional absorption efficiency, maintaining over 95% absorption across a wide spectral range. The absorber’s polarization insensitivity and thermal stability make it a promising candidate for photovoltaics and other energy conversion applications. (Contribution 4).
2. Photocatalytic water splitting with GeC/MXY heterojunctions.
  GeC/MXY (M= Zr, Hf; X,Y= S, Se) heterojunctions were explored for water splitting. These structures demonstrated strong light-harvesting capabilities, small bandgaps, and efficient charge carrier separation due to built-in electric fields at the heterointerface. This research emphasizes the potential of 2D materials for producing clean hydrogen energy. (Contribution 5).
3. Catalysts for hydrogen evolution reaction.
  A gold nanoparticle–mesoporous carbon composite was developed as a catalyst for hydrogen evolution. The material achieved high stability and low activation energy, making it an efficient solution for sustainable hydrogen production. This work exemplifies the importance of 2D material composites in addressing the growing energy demands. (Contribution 6).
4. Pt-Pd nanoparticles for fuel cells.
  Pt-Pd alloy nanoparticles anchored on graphene nanoplates demonstrated enhanced mass activity and durability in oxygen reduction reactions. These materials hold significant promise for advancing proton exchange membrane fuel cells, a key technology for clean energy systems. (Contribution 7).

## 4. Environmental and Catalytic Applications

The environmental impact of industrial processes and the need for efficient catalysis have spurred interest in 2D materials. This SI includes three contributions to addressing these challenges.

1. Tribocatalytic degradation of antibiotics.
  Rare-earth-modified zinc oxide powders were shown to degrade tetracycline antibiotics using triboelectric effects. This approach converts mechanical energy into catalytic activity, providing a sustainable method for water purification and pollution control. (Contribution 8).
2. Gas desulfurization with recycled graphite.
  Recycled graphite from spent Zn/C batteries was transformed into reduced GO-based sorbents. These materials demonstrated competitive desulfurization performance, offering a cost-effective and environmentally friendly alternative to commercial sorbents. (Contribution 9).
3. Ethylene adsorption for agricultural applications.
  Activated carbons derived from agricultural waste were impregnated with copper oxide, significantly improving ethylene adsorption. This innovation has practical applications in prolonging the post-harvest life of fruits and vegetables, showcasing the versatility of 2D materials in agriculture. (Contribution 10).

## 5. Sensors and Optoelectronics

The unique electronic and optical properties of 2D materials enable their application in advanced sensing and optoelectronic devices. This SI includes two contributions to the development and design of high-performance optoelectronic devices.

1. Graphene nanoplatelets for optoelectronics.
  Graphene nanoplatelets integrated with amorphous germanium substrates exhibited enhanced optical absorption and increased refractive indices. These findings pave the way for the development of high-performance optoelectronic devices, such as photodetectors and solar cells. (Contribution 11).
2. Modified quantum dots for optical devices.
  Theoretical studies on GaAs quantum dots with modified confining potentials revealed tunable optical absorption coefficients. This study contributes to the design of new optoelectronic devices leveraging inter-sub-band transitions. (Contribution 12).

## 6. Theoretical Insights and Fundamental Studies

Theoretical studies provide critical insights into the behavior and design of 2D materials, guiding experimental efforts. This SI includes three contributions to the DFT calculations for the 2D materials.

1. Topological insulators and magnetic proximity.
  Bi_2_Se_3_/CrWI_6_ heterostructure was studied to achieve magnetic proximity-induced spin splitting, enabling the quantum anomalous Hall effect. This study advances the field of topological insulators, essential for quantum computing and spintronics. (Contribution 13).
2. Cation–π interactions in graphene.
  Pi-stacked host molecules were synthesized to study their interactions with metal cations. The results demonstrated enhanced binding in stacked configurations, informing the design of materials for ion intercalation and energy storage. (Contribution 14).
3. Corrosion potentials of zinc alloys.
  Using DFT simulations, researchers analyzed the corrosion behavior of zinc alloys in various harsh environments. The findings provide valuable indicators for developing biodegradable metals and corrosion-resistant coatings. (Contribution 15).

## 7. Advanced Functional Materials

Novel functional materials were developed for targeted applications, including ion separation and chemical catalysis. This SI consists of two contributions discussing these applications.

1. GO/MXene composite membranes for ion separation.
  Polyethyleneimine-coated GO/MXene membranes exhibited high efficiency in separating Mg^2+^ and Li^+^ ions from salt lake brines. This technology addresses the critical need for lithium extraction in renewable energy systems. (Contribution 16).
2. Biomass-derived catalysts for sulfonylation.
  A biomass-derived copper catalyst enabled efficient sulfonylation of aniline derivatives, demonstrating a recyclable and sustainable approach to heterogeneous catalysis. (Contribution 17).

## 8. Conclusions

The 17 papers in this SI of *Molecules* exemplify the versatility and transformative potential of 2D materials. From cutting-edge synthesis techniques to innovative applications in energy, environment, and technology, these studies demonstrate the profound impact of 2D materials on addressing global challenges [4].

The integration of experimental advancements with theoretical insights will undoubtedly drive future breakthroughs. As researchers continue to explore the possibilities of 2D materials, their role in shaping sustainable and efficient technologies will only expand [5]. This collection not only highlights current progress, but also sets the stage for a future where 2D materials redefine the boundaries of science and engineering. We sincerely hope the inspirations will contribute to the next edition of this SI, “Two-Dimensional Materials: From Synthesis to Applications, Second Edition” [6].

## Abbreviations

The following abbreviations are used in this manuscript:

2D	Two-dimensional
GO	Graphene oxide
SI	Special Issue
VMSF	Vertically ordered mesoporous silica films
DFT	Density functional theory

## References

[1] Avouris P., Heinz T.F., Low T. (2017). 2D Materials: Properties and Devices.

[2] Tian H., Ren C., Wang S. (2022). Valleytronics in two-dimensional materials with line defect. Nanotechnology.

[3] Yang E.H. (2020). Synthesis, Modelling and Characterization of 2D Materials and Their Heterostructures.

[4] Enoki T., Ando T. (2020). Physics and Chemistry of Graphene: Graphene to Nanographene.

[5] Anasori B., Gogotsi Y. (2019). 2D Metal Carbides and Nitrides (MXenes): Structure, Properties and Applications.

[6] Molecules|Special Issue: Two-Dimensional Materials: From Synthesis to Applications, 2nd Edition. https://www.mdpi.com/journal/molecules/special_issues/VY337364WE.

